# Size-Specific Predictors for Malignancy Risk in Follicular Thyroid Neoplasms: Machine Learning Analysis

**DOI:** 10.2196/73069

**Published:** 2025-07-11

**Authors:** Xin Li, Wen-yu Yang, Fan Zhang, Rui Shan, Fang Mei, Shi-Bing Song, Bang-Kai Sun, Jing Chen, Run-ze Hu, Yang Yang, Yi-hang Yang, Jing-yao Liu, Chun-Hui Yuan, Zheng Liu

**Affiliations:** 1 Department of General Surgery, Peking University Third Hospital, Beijing, China; 2 China Center for Health Development Studies, Peking University, Beijing, China; 3 Department of Ultrasound, Peking University Third Hospital, Beijing, China; 4 Department of Maternal and Child Health, School of Public Health, Peking University, 38 Huayuan Road, Haidian District, Beijing, Beijing, 100191, China, 86 82801222; 5 Department of Pathology, Peking University Third Hospital, School of Basic Medical Sciences, Peking University Health Science Center, Beijing, China; 6 Information Management and Big Data Center, Peking University Third Hosptial, Beijing, China

**Keywords:** follicular thyroid neoplasm, tumor size, machine learning, malignancy, follicular thyroid cancer, follicular thyroid adenoma, random forest, XGBoost

## Abstract

**Background:**

Surgeons often face challenges in distinguishing between benign and malignant follicular thyroid neoplasms (FTNs), particularly small tumors, until diagnostic surgery is performed.

**Objective:**

This study aimed to identify the size-specific predictors for the malignancy risk of FTNs preoperatively.

**Methods:**

A retrospective cohort study was conducted at Peking University Third Hospital in Beijing, China, from 2012 to 2023. Patients with a postoperative pathological diagnosis of follicular thyroid adenoma (FTA) or follicular thyroid carcinoma (FTC) were included. FTNs were classified into small- and large-sized categories based on the cutoff value of the tumor diameter derived from spline regression, which indicated the turning point of malignancy risk. We identified the 5 most important predictors from 22 variables including demography, sonography, and hormones, using machine learning methods. We also calculated the odds ratios (OR) with 95% CI for these predictors in both small- and large-sized FTNs.

**Results:**

Altogether, we included 1494 FTNs, comprising 1266 FTAs and 228 FTCs. FTNs with a maximum diameter less than 3.0 cm were grouped as small-sized tumors (n=715), while those with larger diameters were categorized as large-sized tumors (n=779). In the small-sized group, tumors with macrocalcification (OR 2.90, 95% CI 1.50-5.60), those with peripheral calcification (OR 4.50, 95% CI 1.50-13.00), and those in younger patients (OR 1.33, 95% CI 1.05-1.69) showed a higher malignancy risk. In the large-sized group, tumors presenting with a nodule-in-nodule appearance (OR 3.30, 95% CI 1.30-7.90) exhibited a higher malignancy risk. In both groups, lower thyroid-stimulating hormone levels (OR 1.49, 95% CI 1.20-1.85 for small-sized FTNs; OR 1.61, 95% CI 1.37-1.96 for large-sized FTNs) and a larger mean diameter (OR 1.40, 95% CI 1.10-1.70 for small-sized FTNs; OR 1.50 95% CI 1.20-1.70 for large-sized FTNs) were associated with the malignancy risk of FTNs.

**Conclusion:**

This study identified size-specific predictors for malignancy risk in FTNs, highlighting the importance of stratified prediction based on tumor size.

## Introduction

In recent years, the incidence of thyroid cancer has been growing fast [1], and it is expected to continue to increase in a pronounced manner; the 5-year average annual percent change in the incidence of thyroid neoplasms from 1983 to 2017 in China was 7.82% in men and 8.59% in women [2]. Follicular thyroid neoplasms (FTNs) are one of the most important types of thyroid tumors in addition to papillary thyroid tumors; FTNs account for approximately 10‐15% of all thyroid cancers [3]. Notably, FTNs are much more challenging for clinical management compared with papillary thyroid tumors [4,5]. This situation results from the fact that over 95% of FTNs cannot be accurately diagnosed as benign or malignant, regardless of using ultrasound, cytology, or biomarkers. Currently, clinicians often use diagnostic surgery to distinguish between benign and malignant FTNs. Still, this might lead to unnecessary diagnostic surgery for patients finally diagnosed with the benign type of FTN (ie, follicular thyroid adenoma (FTA]) or a second surgery after the initial diagnostic surgery for those finally diagnosed with the malignant type of FTN (ie, follicular thyroid carcinoma (FTC]) [6]. It is thus crucial to improve the accuracy for the prediction of the malignancy risk of FTNs prior to the diagnostic surgery, which could not only avoid the unnecessary diagnostic surgery for patients with FTA, but also provide timely clinical decisions for patients with FTC.

In the clinical context, three approaches are typically used to predict the malignancy risk of FTNs before the diagnostic surgery: cytology, biomarkers, and ultrasound. Concerning cytology, neither fine needle aspiration cytopathology nor core needle histopathology can reliably differentiate FTA from FTC. The reason is that the pathological diagnosis of FTN requires comprehensive sampling of the entire tumor following surgical dissection to determine the presence of capsular or vascular invasion across all tumor margins. Regarding the use of biomarkers, the routine clinical application, in most cases, is still hindered by the cost-ineffectiveness of testing, as well as suboptimal predictive performance in terms of sensitivity and specificity. Therefore, the ultrasound examination of FTNs is of great significance in facilitating the evaluation of the malignancy risk of FTNs and the necessity for further diagnostic surgery.

Despite considerable efforts to preoperatively distinguish between FTA and FTC, research gaps remained, particularly concerning small-sized FTNs [7-10]. This is attributable to the fact that, on average, FTC exhibits a larger tumor diameter compared to FTA [10,11]; nevertheless, clinicians have reported the existence of small-sized FTC in routine clinical care [12]. If the suboptimal performance of existing prediction models for small-sized FTNs remains inadequately elucidated [13], there would continue to be a significant risk of misdiagnosis and undertreatment of small-sized FTNs. Consequently, it is imperative to identify important predictors (especially those from ultrasound examination) associated with the malignancy risk of both large- and small-sized FTNs.

Our study aimed to (1) use machine learning to identify crucial predictors for the malignancy risk of both small- and large-sized FTNs, and (2) compare the differences in the direction and magnitude of predictors between the small- and large-sized FTNs. Findings from our study would facilitate the precision of differentiating between benign and malignant FTNs.

## Methods

### Overview

**Figure 1.**
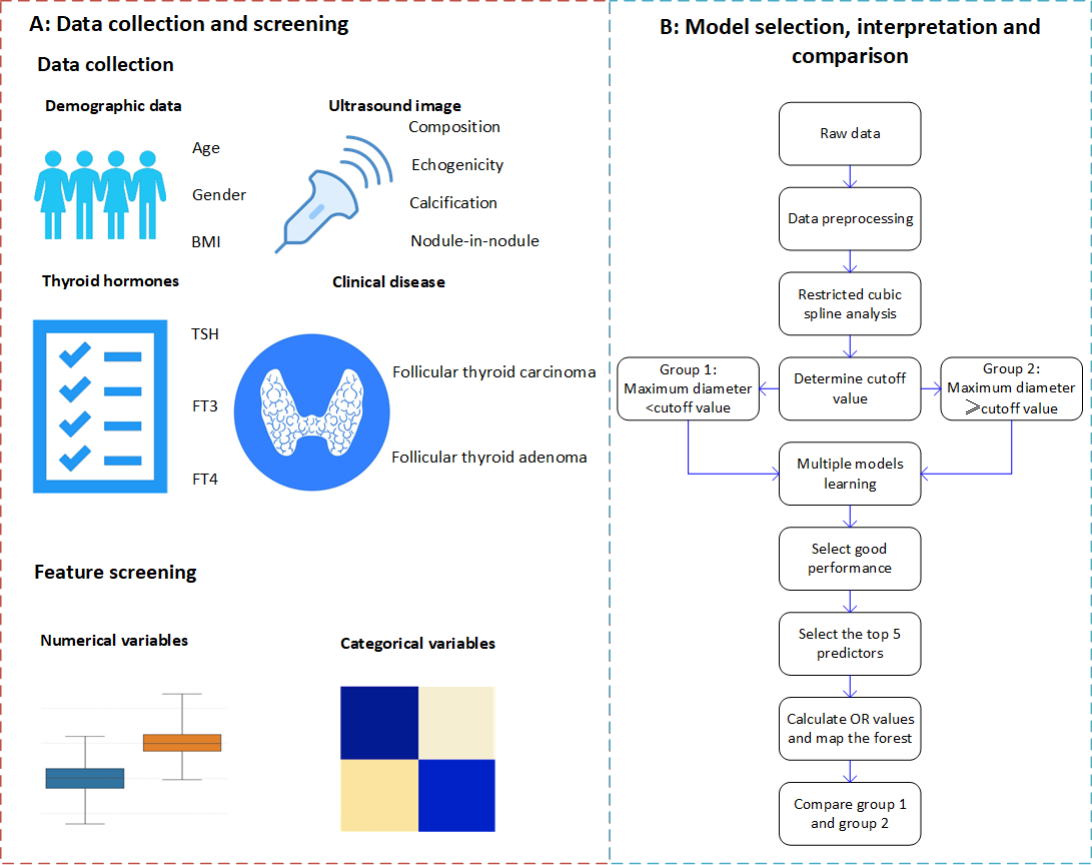
Framework of the study. FT3: free triiodothyronine; FT4: free thyroxine; TSH: thyroid-stimulating hormone.

We reported this retrospective cohort study following the suggestion of the TRIPOD [14] (Transparent Reporting of a multivariable prediction model for Individual Prognosis or Diagnosis Statement), TRIPOD-AI [15] (Transparent Reporting of a multivariable prediction model for Individual Prognosis Or Diagnosis-Artificial Intelligence Statement), and Guidelines for Developing and Reporting Machine Learning Predictive Models in Biomedical Research [16] (Multimedia Appendix 1). The framework of the study is shown in Figure 1.

### Study Population

Our research team included multidisciplinary experts in the field of epidemiology, pathology, ultrasound, surgery, and endocrinology. We conducted a retrospective cohort study at Peking University Third Hospital in Beijing, China, from January 2012 to September 2023. The primary data source for this retrospective cohort study was the electronic medical record system of Peking University Third Hospital. Patients were included if they were pathologically diagnosed with FTC or FTA after surgical treatment; patients were excluded if they did not have ultrasound examinations before surgery. To ensure the accuracy of the pathological diagnosis of FTN, we invited the experienced pathologists to double-check all the pathological diagnoses of FTN based on the 2022, 5th edition WHO Classification of Thyroid Neoplasm [17].

### Classification of FTNs Into Small- and Large-Sized Categories

We first scanned the entire dataset to identify and remove duplicate patient records by using a combination of unique identifiers such as patient ID, date of surgery, and specific pathological report numbers. For categorical variables with a relatively low proportion (5% or less) of missing values, we used imputation based on the majority class. For numerical variables such as patient age or tumor size, we used mean imputation. To classify the FTNs into small- and large-sized categories, we developed a restricted cubic spline model to identify the cutoff value. Specifically, we used the maximum tumor diameter as the continuous variable (predictor); the malignancy risk of FTNs as the outcome; and the covariates included composition, echogenicity, margin, halo, taller-than-wide, calcification, internal blood flow, vascularity, trabecular formation, nodule-in-nodule appearance, mean diameter, thyroid-stimulating hormone (TSH), free triiodothyronine (FT3), free thyroxine (FT4), mean TSH score (interval-adjusted detailed TSH score [18]), tRMSSD of TSH (the time-adjusted root mean square of successive differences of TSH [19]), mean TSH (mean value of preoperative TSH), and coefficient of variation of TSH (the ratio of the SD of preoperative TSH to the mean value of preoperative TSH). To determine the optimal number of nodes for the restricted cubic splines, we employed the Akaike information criterion to strike the balance between the model goodness of fit and complexity that most effectively aligns with the data [20]. Finally, we divided FTNs into two groups: small- (maximum diameter less than the cutoff value) and large-sized (maximum diameter greater than the cutoff value) FTNs.

### Predictors for the Malignancy Risk of FTNs

We selected the predictors for the malignancy risk of FTNs based on our domain knowledge [21,22] and data available. The predictors mainly included patients’ gender, age, BMI, ultrasound features, thyroid hormones, and Hashimoto thyroiditis. To ensure the validity of measurements of predictors, both researchers and clinicians carefully checked the data source of the predictors.

Ultrasound features included composition (solid, predominantly solid, predominantly cystic, or cystic), echogenicity (hyperechoic, isoechoic, hypoechoic, or anechoric), margin (circumscribed, ill-defined, irregular, or lobulated), halo (absent halo, even thickness halo, present halo without evenness of thickness reported, or uneven thickness halo), taller-than-wide (absent or present), calcification (no echogenic foci, microcalcification, macrocalcification, peripheral calcification, microcalcification with comet-tail artifacts, or punctate echogenic foci of undetermined significance), internal blood flow (absent or present), vascularity (mainly central vascularity, mainly peripheral vascularity, mixed vascularity, or avascularity), trabecular formation (absent or present), nodule-in-nodule appearance (absent or present), and mean diameter.

The measurements of thyroid hormones included TSH, FT3, FT4, and TSH-related features. As listed in Table S1 in Multimedia Appendix 2, TSH-related features included the mean TSH score, the tRMSSD of TSH, mean TSH, and coefficient of variation of TSH. The diagnostic criteria for Hashimoto thyroiditis referred to the ultrasonography describing the thyroid tissue as substantial diffuse lesions and the pathology report describing the thyroid tissue as Hashimoto thyroiditis.

### Development and Validation of Machine Learning–Based Models

We established the machine learning–based models, as shown in Figure 1. We trained eight classification models including logistic regression, lasso regression, weighted k-nearest neighbor, decision tree, random forest, naive bayes, XGBoost, and support vector machine (SVM). In total, 70% of the dataset was allocated for model training and selection, while the remaining 30% was reserved for internal validation. We developed and validated the models stepwise through predictor preprocessing, model training, hyperparameter tuning, and 5-fold cross-validation. It is important to note that our study population was from the real-world, naturally distributed population so that the outcome variable was slightly imbalanced (approximately 30% was FTC among all types of FTNs). We adopted the synthetic minority over-sampling technique (SMOTE), which increased the sample size of a few classes by creating new synthetic samples rather than simply copying existing ones [23]. We evaluated the model performance using accuracy, F1 score, the area under the receiver operating characteristic curve (AUROC), the area under the precision-recall curve (AUPRC), sensitivity, and specificity. We comprehensively considered the performance and interpretability of models and selected the most suitable model. We used the mlr3 [24] ecosystem in R 4.4.1 to conduct machine learning.

### Comparison of Important Predictors for Malignancy Risk Between Small- and Large-Sized FTNs

We selected important predictors for small- and large-sized FTNs, respectively. We evaluated the importance of predictors (feature importance) by computing the cross-entropy loss (loss: ce) of all features and visualized the importance of features. We identified the first five most important predictors that could both predict the outcome and not overlap with other predictors based on medical expertise and a novel information-gain approach. Based on the concept of entropy from information theory, the information gain approach is used to assess the extent to which features reduce uncertainty or increase the amount of information [25]. This approach helps to determine which features most effectively enhance classification accuracy by calculating the difference in information entropy before and after feature classification [25].‌ After the selection of important predictors, we calculated the odds ratio (OR) with 95% CI and drew the forest maps using multivariate logistic regression models.

### Ethical Considerations

This study was approved by the Medical Research Ethics Committee of Peking University Third Hospital (No. IRB00006761- M2023168). As a retrospective analysis, the study was granted a waiver for additional informed consent. During the data extraction process, strict confidentiality measures were implemented to ensure patient privacy and data security. All extracted data were anonymized, with any information that could directly identify patients being removed.

## Results

### Characteristics of the Study Population and Selection of the Cut-Off Value for the Size of FTNs

**Figure 2.**
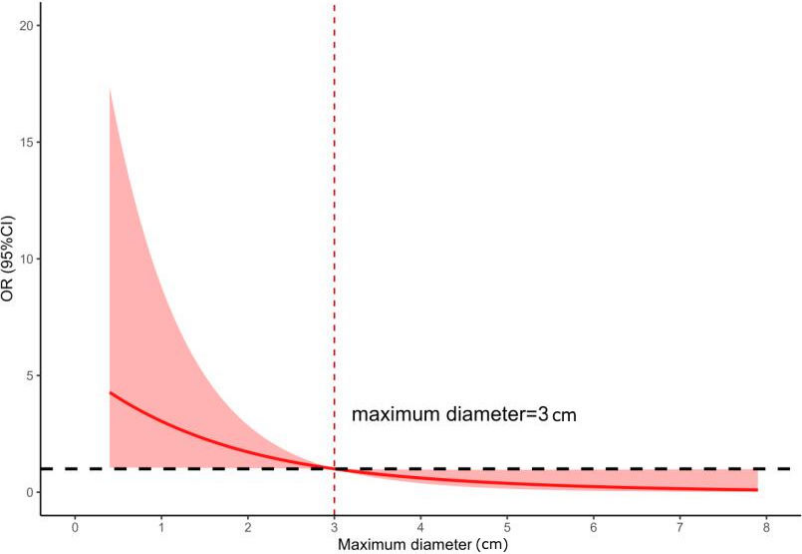
Restricted cubic spline regression analysis of the association between the maximum diameter and the malignancy risk of follicular thyroid neoplasms (FTNs).

Among the included 1494 patients, 1266 (84.7%) were diagnosed with FTA, and the remaining were diagnosed with FTC; the average (SD) age of the patients was 48.25 (0.75) years, and 1127/1494 patients (75.4%) were female. We used a restricted cubic spline model with three nodes that optimally balanced fitting the nonlinear relationship within the data while minimizing the overfitting risk. This analysis revealed a key turning point: the slope of the curve changed distinctly at a maximum diameter of 3 cm for FTNs (Figure 2), indicating that the influence of the maximum diameter on the risk of FTN malignancy shifted at this threshold. As such, 715/1494 tumors (47.9%) were classified as small-sized FTNs (maximum diameter <3 cm), while 779/1494 tumors (52.1%) were large-sized FTNs (maximum diameter ≥3 cm). The characteristics of the study population and FTNs are listed in Tables1  2, respectively.

**Table 1.** Characteristics of the participants having FTNs^a^ with a maximum diameter of <3 cm.

Characteristics	FTA^b^ (n=630)	FTC^c^ (n=85)	Chi-square (*df*)	*P* value
Characteristics of the study population				
Female, n (%)	482 (76.5)	72 (84.7)	*χ*^2 _1_^=2.44	.12^d^
Age (years), mean (SD)	49.20 (13.32)	45.40 (14.75)	*χ*^2 _59_^=63.61	.32^e^
Hashimoto thyroiditis, n (%)	176 (27.9)	32 (37.6)	*χ*^2 _455_^=458.55	.01^d^
BMI (kg/m^2^), mean (SD)	24.16 (3.57)	24.18 (3.52)	*χ*^2 _1_^=2.44	.45^e^
TSH^f^ (µIU/mL), mean (SD)	1.82 (1.14)	2.31 (1.76)	*χ*^2 _275_^=278.65	.43^e^
Mean TSH score^g^, mean (SD)	0.34 (1.19)	−0.26 (1.17)	*χ*^2 _563_^=561.41	.51^e^
tRMSSD of TSH^h^, mean (SD)	0.16 (0.65)	0.12 (0.23)	*χ*^2 _554_^=550.51	.53^e^
Mean TSH^i^, mean (SD)	3.48 (4.94)	2.72 (4.58)	*χ*^2 _533_^=534.06	.48^e^
Coefficient of variation of TSH^j^, mean (SD)	0.78 (0.52)	0.90 (0.56)	*χ*^2 _564_^=562.36	.51^e^
FT3^k^ (pg/mL), mean (SD)	3.23 (0.64)	3.29 (0.52)	*χ*^2 _152_^=172.25	.13^e^
FT4^l^ (ng/dL), mean (SD)	1.28 (0.19)	1.25 (0.22)	*χ*^2 _1_^=2.44	.27^e^
Characteristics of the FTNs, n (%)				
Composition				
Solid	365 (63.5)	50 (63.2)	*χ*^2 _3_^=4.73	.19^m^
Predominantly solid	159 (27.7)	27 (34.2)		
Predominantly cystic	39 (6.8)	1 (1.3)		
Cystic	12 (2.0)	1 (1.3)		
Echogenicity				
Anechoic	5 (0.9)	0 (0.0)	*χ*^2 _3_^=12.71	.006^n^
Hyperechoic	8 (1.4)	4 (4.8)		
Isoechoic	292 (50.1)	28 (33.3)		
Hypoechoic	278 (47.6)	52 (61.9)		
Margin				
Circumscribed	463 (78.7)	46 (56.1)	*χ*^2 _3_^=25.07	<.001^m^
Ill-defined	22 (3.7)	4 (4.9)		
Irregular	72 (12.3)	18 (22.0)		
Lobulated	31 (5.3)	14 (17.0)		
Halo				
Absence of halo	285 (49.2)	36 (46.8)	*χ*^2 _3_^=18.14	<.001^n^
Presence of halo, even thickness	194 (33.5)	20 (26.0)		
Presence of halo, evenness of thickness unknown	33 (5.7)	0 (0.0)		
Presence of halo, uneven thickness	67 (11.6)	21 (27.2)		
Taller-than-wide				
Absence	514 (90.2)	67 (84.8)	*χ*^2 _1_^=2.13	.21^d^
Presence	56 (9.8)	12 (15.2)		
Calcification				
No echogenic foci	488 (77.5)	52 (61.2)	*χ*^2 _5_^=18.25	.004^n^
Microcalcification	59 (9.4)	11 (12.9)		
Macrocalcification	56 (8.9)	15 (17.6)		
Peripheral calcification	12 (1.9)	6 (7.1)		
Microcalcification with comet-tail artifacts	8 (1.2)	1 (1.2)		
Punctate echogenic foci of undetermined significance	7 (1.1)	0 (0.0)		
Internal blood flow				
Absence	97 (16.1)	10 (12.2)	*χ*^2 _1_^=0.83	.36^d^
Presence	506 (83.9)	72 (87.8)		
Vascularity				
Mainly central vascularity	29 (7.2)	5 (7.9)	*χ*^2 _3_^=6.44	.08^n^
Mainly peripheral vascularity	2 (0.5)	0 (0.0)		
Mixed vascularity	219 (54.3)	44 (69.8)		
Avascularity	153 (38.0)	14 (22.3)		
Trabecular formation				
Absence	520 (98.3)	72 (96.0)	*χ*^2 _1_^=0.80	.37^m^
Presence	9 (1.7)	3 (4.0)		
Nodule-in-nodule appearance				
Absence	525 (99.2)	71 (94.7)	*χ*^2 _1_^=7.32	.007^m^
Presence	4 (0.8)	4 (5.3)		
Mean diameter (SD)	1.43 (0.54)	1.56 (0.60)		.09^e^

a FTNs: follicular thyroid neoplasms.

b FTA: follicular thyroid adenoma.

c FTC: follicular thyroid carcinoma.

d Used the Pearson *χ*2 test.

e Used the Kruskal-Wallis test.

f TSH: thyroid-stimulating hormone.

g Mean TSH score: standardized interval-adjusted detailed thyroid-stimulating hormone score.

h TRMSSD of TSH: time-adjusted root mean square of successive differences of thyroid-stimulating hormone.

i Mean TSH: mean value of preoperative TSH.

j Coefficient of variation of TSH: coefficient of variation of thyroid-stimulating hormone.

k FT3: free triiodothyronine.

l FT4: free thyroxine.

m Used the Pearson *χ*2 test with the Yates continuity correction formula.

n Used the Fisher precision probability test.

**Table 2.** Characteristics of participants having FTNs^a^ with a maximum diameter of ≥3 cm.

Characteristics	FTA^b^ (n=636)	FTC^c^ (n=143)	Chi-square (*df*)	*P* value
Characteristics of the study population				
Female, n (%)	478 (75.2)	95 (66.4)	*χ*^2 _1_^=1.81	.18^d^
Age (years), mean (SD)	47.32 (14.81)	49.85 (15.01)	*χ*^2 _64_^=65.58	.42^e^
Hashimoto thyroiditis, n (%)	168 (26.4)	43 (30.1)	*χ*^2 _1_^=2.96	.09^d^
BMI (kg/m^2^), mean (SD)	24.14 (3.78)	24.85 (3.90)	*χ*^2 _495_^=477.31	.71^e^
TSH^f^ (µIU/mL), mean (SD)	1.70 (2.30)	1.86 (1.42)	*χ*^2 _282_^=303.13	.19^e^
Mean TSH score^g^, mean (SD)	0.51 (1.06)	-0.27 (1.27)	*χ*^2 _625_^=627.81	.46^e^
tRMSSD of TSH^h^, mean (SD)	0.14 (0.41)	0.12 (0.26)	*χ*^2 _614_^=617.36	.45^e^
Mean TSH^i^, mean (SD)	3.52 (4.47)	2.62 (4.78)	*χ*^2 _575_^=576.02	.48^e^
Coefficient of variation of TSH^j^, mean (SD)	0.69 (0.46)	0.94 (0.55)	*χ*^2 _628_^=628.80	.48^e^
FT3^k^ (pg/mL), mean (SD)	3.31 (0.67)	3.33 (0.76)	*χ*^2 _170_^=167.87	.53^e^
FT4^l^ (ng/dL), mean (SD)	1.26 (0.22)	1.24 (0.30)	*χ*^2 _106_^=106.28	.47^e^
Characteristics of the FTNs, n (%)				
Composition				
Solid	220 (38.8)	73 (57.5)	*χ*^2 _3_^=17.04	<.001^m^
Predominantly solid	239 (42.2)	43 (33.9)		
Predominantly cystic	105 (18.5)	11 (8.6)		
Cystic	3 (0.5)	0 (0.0)		
Echogenicity				
Anechoic	2 (0.4)	0 (0.0)	*χ*^2 _3_^=8.11	.048^m^
Hyperechoic	19 (3.6)	2 (1.5)		
Isoechoic	311 (58.1)	62 (47.7)		
Hypoechoic	203 (37.9)	66 (50.8)		
Margin				
Circumscribed	508 (87.6)	102 (75.0)	*χ*^2 _3_^=21.10	<.001^n^
Ill-defined	14 (2.4)	1 (0.7)		
Irregular	28 (4.8)	17 (12.5)		
Lobulated	30 (5.2)	16 (11.8)		
Halo				
Absence of halo	244 (42.9)	55 (42.0)	*χ*^2 _3_^=11.81	.008^d^
Presence of halo, even thickness	214 (37.6)	37 (28.2)		
Presence of halo, evenness of thickness unknown	43 (7.5)	9 (6.9)		
Presence of halo, uneven thickness	68 (12.0)	30 (22.9)		
Taller-than-wide				
Absence	541 (85.1)	122 (85.3)	*χ*^2 _1_^=2.70	.10^d^
Presence	9 (1.4)	5 (3.5)		
Calcification				
No echogenic foci	528 (83.0)	103 (72.0)	*χ*^2 _5_^=15.58	.008^m^
Microcalcification	31 (4.9)	13 (9.1)		
Macrocalcification	54 (8.5)	22 (15.4)		
Peripheral calcification	2 (0.3)	2 (1.4)		
Microcalcification with comet-tail artifacts	13 (2.0)	3 (2.1)		
Punctate echogenic foci of undetermined significance	8 (1.3)	0 (0.0)		
Internal blood flow				
Absence	51 (8.4)	9 (6.6)	*χ*^2 _1_^=0.50	.48^d^
Presence	557 (91.6)	128 (93.4)		
Vascularity				
Mainly central vascularity	34 (5.9)	9 (6.6)	*χ*^2 _3_^=7.13	.06^n^
Mainly peripheral vascularity	2 (0.3)	0 (0.0)		
Mixed vascularity	308 (53.6)	89 (65.0)		
Avascularity	231 (40.2)	39 (28.4)		
Trabecular formation				
Absence	587 (96.7)	129 (92.1)	*χ*^2 _1_^=5.95	.02^d^
Presence	20 (3.3)	11 (7.9)		
Nodule-in-nodule appearance				
Absence	593 (97.7)	131 (93.6)	*χ*^2 _1_^=6.48	.01^d^
Presence	14 (2.3)	9 (6.4)		
Mean diameter (SD)	3.23 (0.85)	3.68 (1.09)	*χ*^2 _133_^=143.19	.26^e^

a FTNs: follicular thyroid neoplasms.

b FTA: follicular thyroid adenoma.

c FTC: follicular thyroid carcinoma.

d Used the Pearson *χ*2 test.

e Used the Kruskal-Wallis test.

f TSH: thyroid-stimulating hormone.

g Mean TSH score: standardized interval-adjusted detailed thyroid-stimulating hormone score.

h tRMSSD of TSH: time-adjusted root mean square of successive differences of thyroid-stimulating hormone.

i Mean TSH: mean value of preoperative TSH.

j Coefficient of variation of TSH: coefficient of variation of thyroid-stimulating hormone.

k FT3: free triiodothyronine.

l FT4: free thyroxine.

m Used the Fisher precision probability test.

n Used the Pearson *χ*2 test with the Yates continuity correction formula.

### Distinct Predictors for the Malignancy Risk in Small- and Large-Sized FTNs

We compared the performance in discrimination among the eight models (logistic regression, weighted k-nearest neighbor, lasso regression, decision tree, random forest, naive bayes, XGBoost, and SVM). The XGBoost and random forest models performed broadly better in the small- and large-sized FTN groups, respectively (Table 3, Table S2 in Multimedia Appendix 2).

**Table 3.** Model performance in predicting the malignancy risk of small- and large-sized follicular thyroid neoplasms (FTNs).

Models	Small-sized FTNs (maximum diameter <3 cm)			Large-sized FTNs (maximum diameter ≥3 cm)		
	Accuracy	F1 score	AUPRC^a^	Accuracy	F1 score	AUPRC
Logistic regression	0.654	0.301	0.238	0.701	0.450	0.337
Weighted k-nearest neighbor	0.587	0.233	0.140	0.611	0.380	0.350
Lasso regression	0.752	0.299	0.227	0.669	0.411	0.368
Decision tree	0.614	0.205	0.134	0.646	0.381	0.274
Random forest	0.790	0.286	0.206	0.704	0.459	0.422
Naive bayes	0.577	0.220	0.163	0.623	0.297	0.251
XGBoost	0.811	0.330	0.248	0.695	0.403	0.380
SVM^b^	0.643	0.226	0.151	0.691	0.312	0.199

a AUPRC: area under the precision-recall curve.

b SVM: support vector machine.

**Figure 3.**
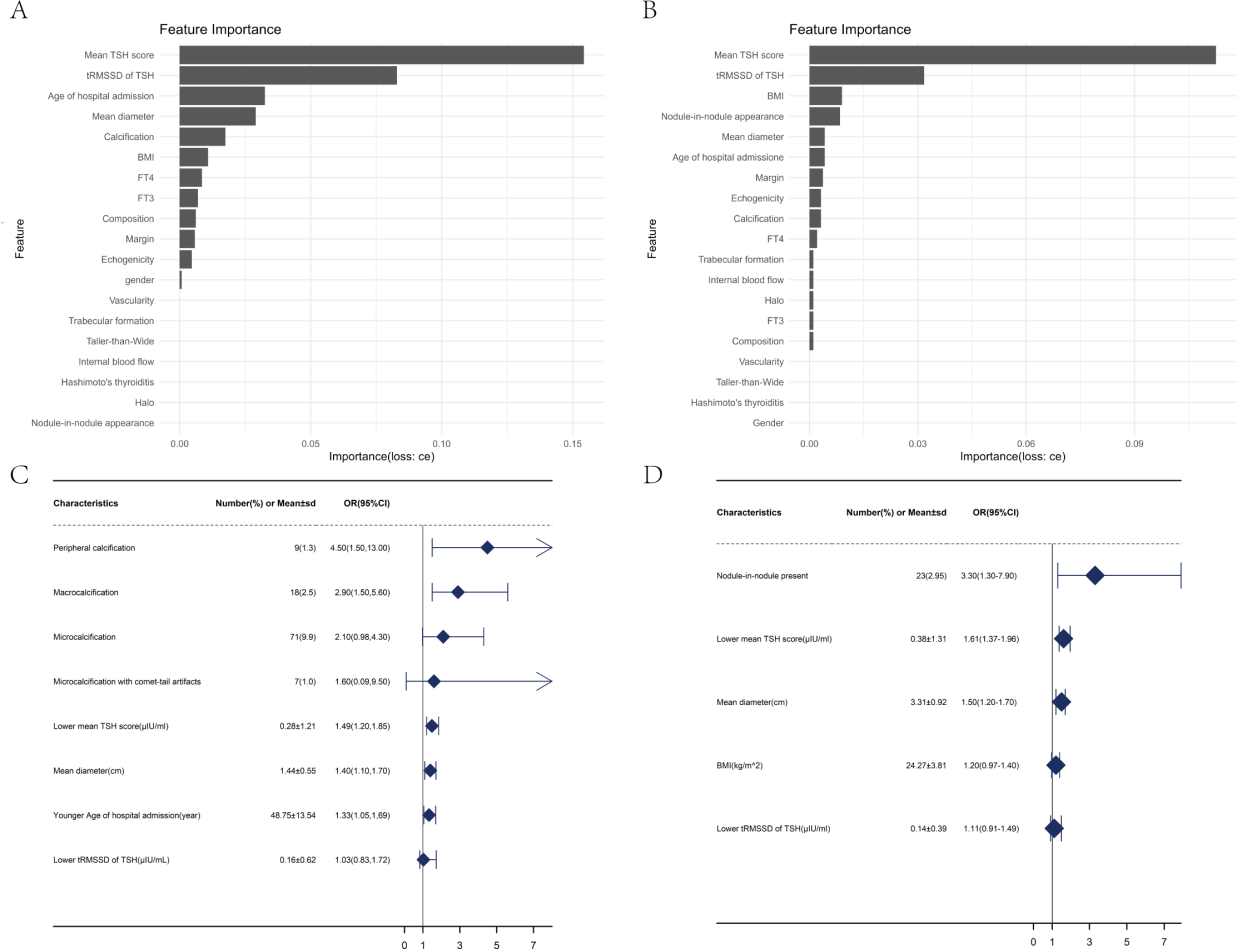
The feature importance and forest map of the top 5 predictors for malignancy risk in small- and large-sized follicular thyroid neoplasms (FTNs). (**A)** The feature importance using the XGBoost model in small-sized follicular tumors, and loss: ce is the cross-entropy loss. (**B)** The feature importance using the random forest model in large-sized follicular tumors, and loss: ce is the cross-entropy loss. (C) The forest map of the top 5 predictors using multivariate logistic regression in small-sized follicular tumors, and the OR and 95% CI of microcalcification, macrocalcification, peripheral calcification, and microcalcification with comet-tail artifacts is based on no computed echogenic foci. (D) The forest map of the top 5 predictors using multivariate logistic regression in large-sized follicular tumors, and the OR and 95% CI of nodule-in-nodule presence is based on the computed nodule-in-nodule absence. FT3: free triiodothyronine; FT4: free thyroxine; tRMSSD: time-adjusted root mean square of successive differences; TSH: thyroid-stimulating hormone.

In the small-sized FTN group, the top five predictors were the mean TSH score, tRMSSD of TSH, age at hospital admission, mean diameter, and calcification in the XGBoost model (Figure 3). Compared to tumors with no echogenic foci in the ultrasound image, the small-sized FTNs expressing microcalcification (OR 2.10, 95% CI 0.98-4.30), macrocalcification (OR 2.90, 95% CI 1.50-5.60), peripheral calcification (OR 4.50, 95% CI 1.50-13.00), and microcalcification with comet-tail artifacts (OR 1.60, 95% CI 0.09-9.50) had a higher risk of malignancy. Additionally, the risk of malignancy was higher in the patients with small-sized FTNs with a lower mean TSH score (OR 1.49, 95% CI 1.20-1.85), lower tRMSSD of TSH (OR 1.03, 95% CI 0.83-1.72), younger patients (OR 1.33, 95% CI 1.05-1.69), and larger mean diameter (OR 1.40, 95% CI 1.10-1.70).

By contrast, in the large-sized FTN group, the top 5 predictors were the mean TSH score, tRMSSD of TSH, BMI, nodule-in-nodule appearance, and mean diameter in the random forest model (Figure 3). The risk of malignancy was higher in the large-sized FTN, which included the lower mean TSH score (OR 1.61, 95% CI 1.37-1.96), lower tRMSSD of TSH (OR 1.11, 95% CI 0.91-1.49), higher BMI (OR 1.20, 95% CI 0.97-1.40), the presence of nodule-in-nodule (OR 3.30, 95% CI 1.30-7.90), and larger mean diameter (OR 1.50, 95% CI 1.20-1.70).

## Discussion

### Principal Findings

Taking advantage of a long-term cohort based on the real-world data, we found distinctive predictors for the malignancy risk of FTNs between the small- and large-sized tumors. Specifically, the tumor’s calcification appearance, mean diameter, and patients’ age were more important in predicting the malignancy risk of small-sized FTNs, whereas the tumor’s nodule-in-nodule appearance and patients’ BMI were more important in that of large-sized FTNs.

### Comparison to Prior Work

It remained unclear regarding the cut-off value of tumor size that indicated the malignancy risk or surgical indications of FTNs. Concerning clinical guidelines, we observed that neither domestic nor international guidelines have recommended a cut-off value of tumor size for surgical treatment in FTNs [26-28]. Regarding existing research, among the 5 studies included in this review, the cut-off value of tumor size used to predict the malignancy risk of FTNs remained contradictor[29-33]. The included studies reported the associations between tumor size and the malignancy risk of FTNs, but all of them lacked a solid basis for their tumor-size classification criteria prior to surgical treatment.

Based on our literature review, previous studies on this topic often employed a one-size-fits-all model to predict the malignancy risk of FTN, without considering the tumor size[34,35]. For example, a retrospective multicenter study developed a prediction model for FTNs using the Thyroid Imaging Reporting and Data System (TI-RADS), and the validation dataset showed that the follicular TI-RADS model improved the performance in differentiating between FTA and FTC (AUROC 0.81) [34]. Additionally, a model combining the prior-based level set method with a deep convolutional neural network achieved an AUROC of 0.913 in distinguishing FTC from FTA using features extracted from ultrasound images [35]. Furthermore, we evaluated the follicular TI-RADS scoring criteria developed by Li et al [34] with our data and found a lower sensitivity (0.044 with a threshold for FTC risk set at >90%, and 0.269 with using a >50% FTC risk threshold) in evaluating the prediction model. These inconsistencies and the reduced sensitivity observed in external datasets may, at least in part, be attributed to the limitation of not considering tumor size when predicting the malignancy risk.

Our findings suggest that clinical differentiation between benign and malignant FTNs should be more precisely tailored according to the size of the FTNs. For example, the nodule-in-nodule appearance, a characteristic feature of FTC, was more frequently observed in larger FTNs [36]. This sign may reflect heterogeneous tumor cell proliferation, a phenomenon more commonly associated with larger tumors. Conversely, the incidence of this sign is relatively low in small-sized FTNs, complicating the identification of small-sized FTCs. Calcification, however, demonstrates a higher predictive value for malignancy in smaller FTNs. Our study focused on commonly used and easily obtainable predictors in clinical practice, including clinical features, TSH levels, and ultrasound characteristics. The limited number of predictors and the straightforward model design enhance its practicality for physicians. This research assists thyroid specialists in customizing predictor selection to assess the malignancy risk in FTNs of different sizes, ultimately improving the accuracy of FTC identification, patient outcomes, and quality of life while reducing postoperative complications.

Our finding also suggests that the mean TSH score ranked among the top 5 predictors for determining malignancy in tumors of both sizes. A cohort study from the EPIC (European Prospective Investigation into Cancer and Nutrition) cohort has revealed a negative association between elevated TSH levels and an increased risk of thyroid cancer [37]. Similarly, Gudmundsson et al [38] propose that low TSH levels may reduce the differentiation of thyroid epithelium, potentially increasing the predisposition to malignant cell transformation.

In evaluating the predictive performance of our machine learning models, we employed metrics distinct from those used in prior studies. For instance, Li et al [34] reported an AUROC of 0.76 for the LASSO regression model (the ratio of FTA to FTC: training set, 2.74; validation set, 3.70), and also in the LASSO regression model, the AUROC reached 0.913 for discriminating FTA from FTC [35] (the ratio of FTA to FTC: 4.00). However, neither study incorporated the F1 score or AUPRC as evaluation metrics. Given the imbalanced nature of our data (FTA to FTC ratio: 7.00 for small follicular tumors and 5.00 for large follicular tumors), AUPRC provides a more informative and intuitive measure of model performance compared to AUROC [39]. Additionally, the F1 score offers a comprehensive evaluation by integrating precision and recall, ensuring a balanced assessment of model performance in the context of skewed category distributions [40].

### Limitations and Strengths

Our study had several strengths. It was among the first to classify FTNs into two subgroups based on tumor diameter for machine learning analysis, revealing significant differences between these subgroups. Additionally, our models benefit from a large sample size, the use of clinically accessible and validated predictors, and a comprehensive evaluation using metrics appropriate for imbalanced data [41]. Importantly, the distribution of FTA and FTC in our study population was fully consistent with that of patients with FTNs in real-world settings, avoiding any exaggeration of sample sizes during model training.

However, our study had certain limitations. First, our classification of small- and large-sized FTNs based on a threshold of 3 cm was determined by our dataset rather than established guideline consensus, which may limit its generalizability to external datasets. We recommend that future clinical guidelines refine specific ultrasound risk indicators for FTNs based on the findings of this study and subsequent related research. Second, while we applied the SMOTE oversampling strategy to address imbalanced data, this approach may introduce bias when predicting new data [42]. Finally, the lack of external validation for our trained model limits our ability to assess its generalizability, potentially affecting its practical applicability and reliability in predicting new cases [43].

### Future Directions

Our study had important clinical implications. In current clinical practice, it primarily relies on the ultrasonographic features of FTNs to assess the tumors’ malignancy risk under the observation period (ie, before surgical treatment). It is thus crucial to emphasize the need for personalized predictive models in FTNs. Achieving this requires a more refined stratification of tumors based on the clinical characteristics of FTNs, which can enhance the customization of predictive models for tumors of individual cases. This tailored approach acknowledges the inherent variability within FTN categories, suggesting that further stratification within FTNs based on tumor size can facilitate more precise diagnostic and treatment decisions.

To advance this research, we intend to conduct a prospective study to validate the accuracy and reliability of the identified predictors in real-world clinical settings. The prospective evidence will enable updates and refinements to existing medical knowledge and practices for managing FTNs. For FTNs with tumor diameters <3 cm, surgical decision-making should prioritize TSH levels, age at admission, tumor diameter, and calcification status. For FTNs ≥3 cm, TSH levels, BMI, nodule-in-nodule architecture, and tumor diameter should guide clinical recommendations. Furthermore, we advocate that professional medical societies and health care organizations collaboratively develop evidence-based practice guidelines integrating these predictors.

### Conclusion

In our study, we identified differences in predictors among follicular tumors of varying sizes. Clinically, these findings emphasize the importance of considering the size during the preoperative diagnosis of benign versus malignant FTNs. We found that both clinical guidelines and the existing research literature have not sufficiently addressed the optimal size of FTNs for surgical intervention or its correlation with malignancy risk. There is a significant research gap in precisely determining the preoperative size-based classification of FTNs. Thus, further investigations are imperative to address this knowledge deficit.

## Supplementary material

10.2196/73069 Multimedia Appendix 1 Guidelines for developing and reporting machine learning predictive models in biomedical research.

10.2196/73069 Multimedia Appendix 2 Supplementary tables.

10.2196/73069 Checklist 1 TRIPOD-AI Checklist.

## Abbreviations

AUPRC: area under precision-recall curve
EPIC: European Prospective Investigation into Cancer and Nutrition
FTA: follicular thyroid adenoma
FTC: follicular thyroid carcinoma
FTNs: follicular thyroid neoplasms
OR: odds ratio
SMOTE: synthetic minority over-sampling technique
SVM: support vector machine
TI-RADS: thyroid imaging reporting and data system
tRMSSD: time-adjusted root mean square of successive difference
TSH: thyroid-stimulating hormone
