# Supplementary material for: Size-Specific Predictors for Malignancy Risk in Follicular Thyroid Neoplasms: Machine Learning Analysis
Source: JMIR Cancer. 2025 Jul 11;11:e73069. doi: 10.2196/73069 (PMC12274017; doi:10.2196/73069)
Supplement: Multimedia Appendix 1 [file cancer-v11-e73069-s001.docx]

**Consolidated reporting guidelines for prognostic and diagnostic machine learning modeling studies**

**Multimedia Appendix 1: Author Checklist**

The following is the reporting checklist. A response should indicate whether the particular item is documented in the study. If the response to an item is Y then the location in the article should be provided (e.g., section number), and if the response is N or NA then some reasoning should be provided.

| **#** | **Item** | **Y** | **N** | **NA** | **Location / Reasoning** |
| --- | --- | --- | --- | --- | --- |
| **Study Details** | | | | | |
| 1.1 | *The medical/clinical task of interest* | Y |  |  | Page 4-5 |
| 1.2 | *The research question* | Y |  |  | Page 5 |
| 1.3 | *Current medical/clinical practice* | Y |  |  | Page 4-5 |
| 1.4 | *The known predictors and confounders to what is being predicted / diagnosed* | Y |  |  | Page 7-8 |
| 1.5 | *The overall study design* | Y |  |  | Page 6 |
| 1.6 | *The medical institutional setting(s)* |  | N |  |  |
| 1.7 | *The target patient population* | Y |  |  | Page 6 |
| 1.8 | *The intended use of the ML model* | Y |  |  | Page 8 |
| 1.9 | *Existing model performance benchmarks for this task* | Y |  |  | Page 18-19 |
| 1.10 | *Ethical and other regulatory approvals obtained* | Y |  |  | Page 8-9 |
| **The Data** | | | | | |
| 2.1 | *Inclusion / exclusion criteria for the patient cohort* | Y |  |  | Page 5 |
| 2.2 | *Methods of data collection* | Y |  |  | Page 5 |
| 2.3 | *Bias introduced due to the method of data collection used* | Y |  |  | Page 5 |
| 2.4 | *Data characteristics* | Y |  |  | Page 11-14 |
| 2.5 | *Methods of data transformations and preprocessing applied* | Y |  |  | Page 6 |
| 2.6 | *Known quality issues with the data* |  | N |  |  |
| 2.7 | *Sample size calculation* |  | N |  |  |
| 2.8 | *Data Availability* | Y |  |  | Page 20 |
| **Methodology** | | | | | |
| 3.1 | *Strategies for handling missing data* | Y |  |  | Page 6 |
| 3.2 | *Strategies for addressing class imbalance* | Y |  |  | Page 7 |
| 3.3 | *Strategies for reducing dimensionality of data* |  | N |  |  |
| 3.4 | *Strategies for handling outliers* | Y |  |  | Page 6 |
| 3.5 | *Strategies for data augmentation* | Y |  |  | Page 7 |
| 3.6 | *Strategies for model pre-training* | Y |  |  | Page 7-8 |
| 3.7 | *The rationale for selecting the machine learning algorithm* |  | N |  |  |
| 3.8 | *The method of evaluating model performance during training* | Y |  |  | Page 8 |
| 3.9 | *The method used for hyperparameter tuning* | Y |  |  | Page 8 |
| 3.10 | *Model’s output adjustments* |  | N |  |  |
| **Evaluation** | | | | | |
| 4.1 | *Performance metrics used to evaluate the model* | Y |  |  | Page 8 |
| 4.2 | *The cost or consequence of errors* |  | N |  |  |
| 4.3 | *The results of internal validation* | Y |  |  | Page 14 |
| 4.4 | *The final model hyperparameters* |  | N |  |  |
| 4.5 | *Model evaluation on an external dataset* |  | N |  |  |
| 4.6 | *Characteristics relevant for detecting data shift and drift* |  | N |  |  |
| **Explainability and Transparency** | | | | | |
| 5.1 | *The most important features and how they relate to the outcome(s)* | Y |  |  | Page 14 |
| 5.2 | *Plausibility of model outputs* |  | N |  |  |
| 5.3 | *Interpretation of model's results by an end-user* | Y |  |  | Page 14-15 |
