# Supplementary material for: Size-Specific Predictors for Malignancy Risk in Follicular Thyroid Neoplasms: Machine Learning Analysis
Source: JMIR Cancer. 2025 Jul 11;11:e73069. doi: 10.2196/73069 (PMC12274017; doi:10.2196/73069)
Supplement: Multimedia Appendix 2 [file cancer-v11-e73069-s002.docx]

**Supplemental Table 1 Calculation method of TSH related feature**

| TSH related feature | Calculation method |
| --- | --- |
| mean TSH score | $mean TSH score=\sum_{1}^{n} \frac{{TS}_{n}+{TS}_{n+1}}{2}\times\frac{{ID}_{n}}{\sum_{1}^{n} ID}$ |
| tRMSSD of TSH | $tRMSSD of TSH=\sqrt{\frac{1}{N}\sum_{i=1}^{N-1} {(\frac{C_{i+1}-C_{i}}{t_{i+1}-t_{i}})}^{2}}$ |
| mean TSH | $mean TSH=\frac{\sum C_{i}}{n}$ |
| Coefficient of variation of TSH | $Coefficient of variation of TSH=\frac{\sum_{i}^{n} (C_{i}-\frac{\sum C_{i}}{n})}{n-1}/\frac{\sum C_{i}}{n}$ |
| Notes: ID_n_ is each interval (days) and TS_n_ and TS_n+1_ are the TSH scores from the beginning and end of each interval, respectively; N is the total number of TSH data points, C_i_ is the TSH value of the i-th data point, and t_i_ is the time of the i-th data point; n is the total number of TSH data points before operation | |

**Supplemental Table 2 Other index of model performance in FTNS**

| Models | FTNs^a^ with diameter < 3.0cm | | | FTNs^a^ with diameter≥ 3.0cm | | |
| --- | --- | --- | --- | --- | --- | --- |
|  | AUROC^b^ | Sensitivity | Specificity | AUROC^b^ | Sensitivity | Specificity |
| Logistic regression | 0.649 | 0.588 | 0.687 | 0.804 | 0.684 | 0.717 |
| Weighted k-nearest neighbor | 0.593 | 0.500 | 0.623 | 0.767 | 0.649 | 0.602 |
| Lasso regression | 0.722 | 0.647 | 0.639 | 0.715 | 0.632 | 0.677 |
| Decision tree | 0.578 | 0.382 | 0.683 | 0.672 | 0.649 | 0.606 |
| Random forest | 0.708 | 0.353 | 0.849 | 0.742 | 0.684 | 0.709 |
| Naive bayes | 0.602 | 0.941 | 0.107 | 0.598 | 0.526 | 0.547 |
| XGboost | 0.700 | 0.441 | 0.833 | 0.780 | 0.561 | 0.724 |
| SVM | 0.589 | 0.529 | 0.575 | 0.594 | 1.000 | 0.008 |
| ^a^FTNs: Follicular thyroid neoplasms  ^b^AUPRC: Area under precision-recall curve | | | | | | |
